# Relapse of Non-Hodgkin's Lymphoma Involving the Trachea: Acute Subglottic Obstruction

**DOI:** 10.1155/2014/230682

**Published:** 2014-03-04

**Authors:** Allen Y. Wang, Jeffrey T. Wang, Yi Shen, Brett Levin

**Affiliations:** ^1^Department of Otolaryngology, Westmead Hospital, University of Sydney, Westmead, Sydney, NSW 2145, Australia; ^2^Departments of Otolaryngology and Head & Neck, Ningbo Lihuili Hospital, Ningbo Medical Centre, Ningbo, Zhejiang 315041, China

## Abstract

Relapsing lymphoma involving the trachea causing tracheal obstruction is exceedingly uncommon. Despite its rarity, it should be considered in the differential diagnosis when a patient with known lymphoma presents with signs of airway obstruction such as stridor. We report an unusual case of relapsing non-Hodgkin's lymphoma with tracheal involvement in a 57-year-old female and review the relevant literature. It is highly unusual for relapsing lymphoma to involve the trachea causing tracheal obstruction. Despite its rarity, it can present with life-threatening airway obstruction which may be rapidly progressive requiring immediate surgical intervention such as tracheostomy.

## 1. Introduction

The most common etiologies of acute upper airway obstruction include infection, neoplasms, trauma, foreign bodies, and angioedema. Tracheal neoplasm accounts for less than 0.1% of all malignancies [1]. Primary malignant tumors of the trachea are uncommon. The most prevalent histologies in adult primary tracheal tumors are squamous cell carcinoma and adenoid cystic carcinoma [2]. Although extranodal lymphoma is reasonably common, a primary presentation of extranodal lymphoma involving the trachea is extremely unusual. Relapsing lymphoma of the trachea is even more rarely encountered.

In non-Hodgkin's lymphoma, diffuse large B-cell lymphoma is the most common subtype which accounts for approximately 25% of cases [3]. The incidence of diffuse large B-cell lymphoma is approximately 7 cases per 100,000 people [4]. Relapse typically occurs in the first 2-3 years after treatment and usually occurs at a different site which is separate from the primary presentation [5].

We report the first case in the literature of relapsing non-Hodgkin's lymphoma in the trachea who presented with an acutely threatened airway requiring immediate surgical tracheostomy.

## 2. Case Report

A 57-year-old female with a background of B-cell non-Hodgkin's lymphoma in remission presented to the emergency department with rapidly progressive noisy breathing and dyspnoea that was worse when supine over the last 7 days. There were associated symptoms of cough and hemoptysis as well as constitutional symptoms including fever, night sweats, and weight loss over this period. She denied voice changes, dysphagia, odynophagia, or trismus.

The patient was originally diagnosed with B-cell non-Hodgkin's lymphoma 6 months prior and had undergone six cycles of chemotherapy according to the German Multicenter Study Group for Adult Acute Lymphoblastic Leukemia (GMALL) 2002 protocol.

**Figure 1:**
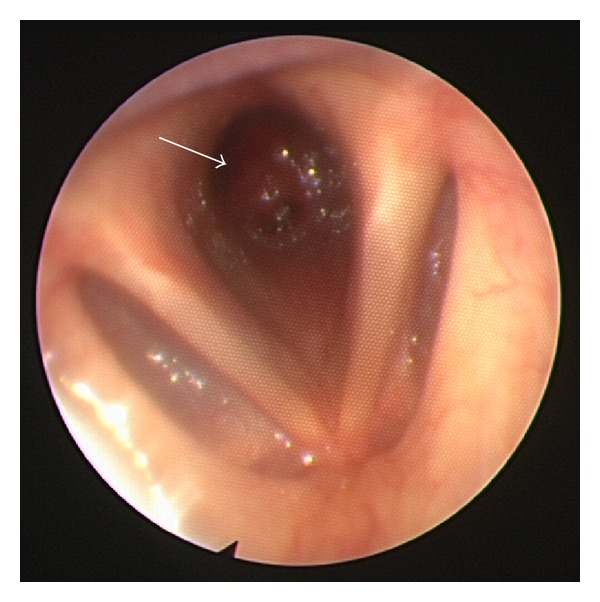
Nasal endoscopy revealed a subglottic mass (arrow) approximately 1 cm in diameter causing near-complete obstruction of the airway.

**Figure 2:**
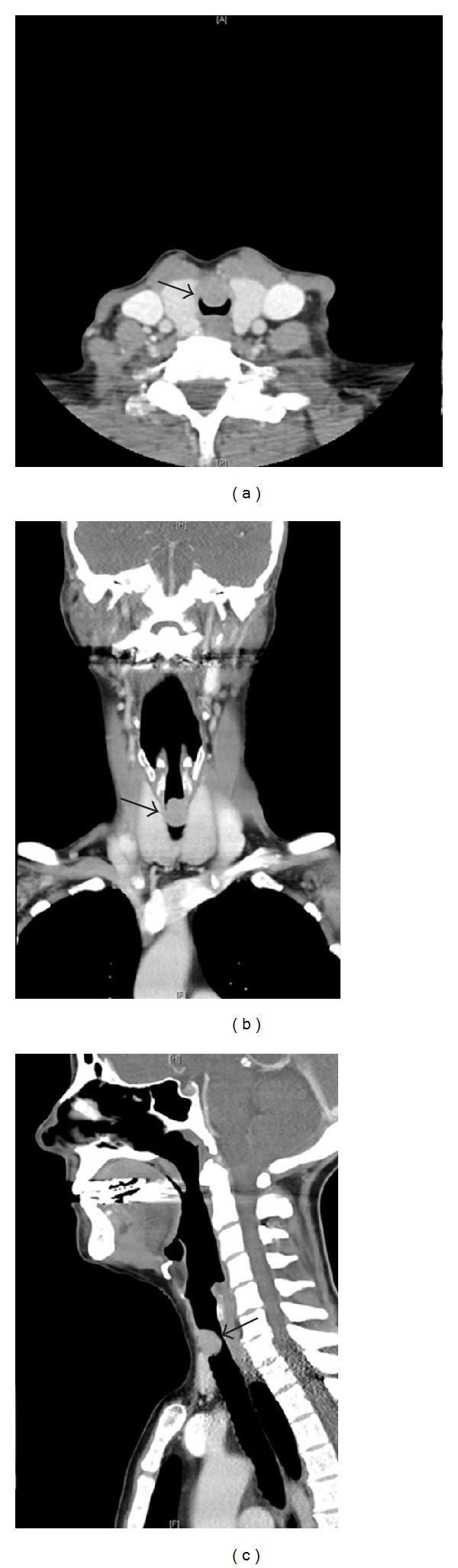
(a) CT neck with intravenous contrast in axial plane, (b) coronal plane, and (c) sagittal plane demonstrating a 1.3 × 1.4 × 1.9 cm round soft tissue mass (arrow) extending posteriorly from the anterior wall of the upper trachea at the level of C6, approximately 2 cm inferior to the true vocal cords associated with significant (>80%) airway obstruction.

On review, she was afebrile with audible biphasic stridor. Both the stridor and dyspnea were significantly exacerbated in the supine position. On nasal endoscopy, there was a large round subglottic mass causing near-complete (>80%) luminal obstruction of the airway (Figure 1). Chest X-ray was unremarkable with clear lung fields. Computed tomography (CT) imaging of her neck demonstrated a round subglottic soft tissue mass (1.3 × 1.4 × 1.9 cm) arising from the anterior wall of the upper trachea at the level of C6 (Figure 2).

**Figure 3:**
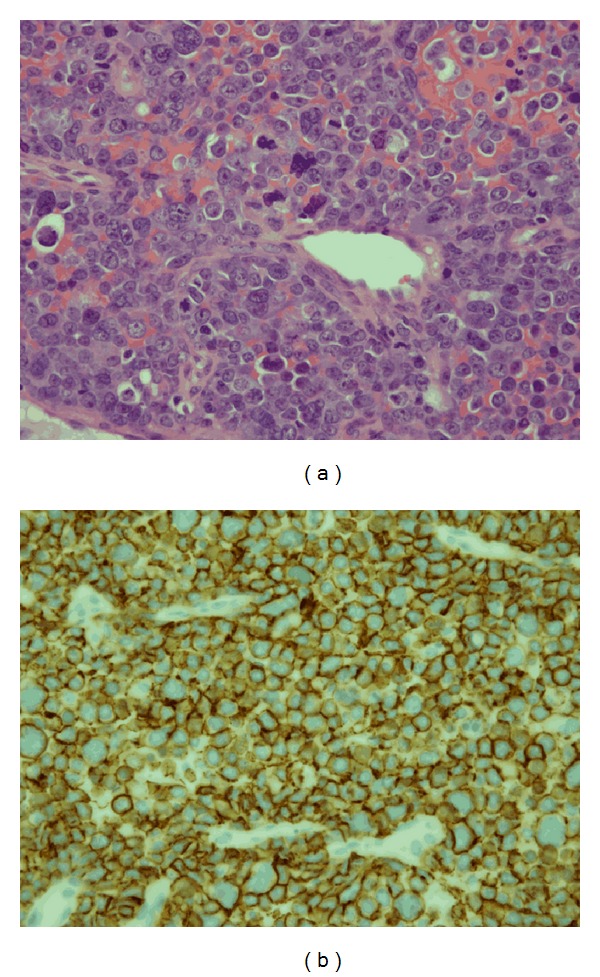
(a) Histopathological image of the biopsied tracheal mass shows an infiltrate of monotonous population of large cells with vesicular nuclei and prominent central nucleoli. Numerous highly anaplastic cells are also seen (H&E, ×40). (b) On immunoperoxidase staining, cells show positive staining for CD 20 confirming malignant B lymphocytes (CD 20, ×40).

Her airway was urgently secured with an awake tracheostomy in the operating theatre, followed by microlaryngoscopy, esophagoscopy, and bronchoscopy. The large flesh-colored subglottic mass was biopsied and debulked with a laryngeal Skimmer Blade (15 degree, 3.5 mm diameter). There were no other synchronous tracheal tumors or upper esophageal lesions. Pathology from the tracheal mass revealed cells staining positive for CD 20 (Figure 3) and MUM 1 and negative for CD 10, BCL 6, and CD 30. Overall, the features were consistent with diffuse large B-cell lymphoma of activated B-cell phenotype. Bone marrow biopsy was negative. Whole body positron emission tomography (PET) scanning revealed multiple areas of active disease, including a high metabolic activity in the subglottis, suggesting recurrent disease.

Six days later, a second operation was performed for endoscopic evaluation and change of tracheostomy tube. Intraoperatively, the debulked tumor was noted to have rapidly regrown, reaching its original size, with evidence of distal extension, confirming its aggressive nature. The patient underwent palliative chemotherapy (procarbazine, gemcytabine, and dexamethasone) and radiotherapy with clinical and radiological remission at 4 months. Whilst she met all decannulation parameters, she remained tracheostomized due the aggressive nature of the disease and high risk of recurrence.

## 3. Discussion

Even though extranodal lymphoma is reasonably common (e.g., in the gastrointestinal tract and the head and neck region), a primary presentation of extranodal lymphoma involving the trachea is extremely unusual. Seven series with a total of 425 tracheal tumors between 1930 and 1989 reported only 1 case of non-Hodgkin's lymphoma (prevalence of 0.23%) [10]. Relapsing lymphoma in the trachea is even more rarely encountered.

Tracheal lymphoma can cause symptoms and signs of upper airway obstruction (e.g., dyspnea and stridor), mucosal ulceration, and irritation (e.g., cough). Given the submucosal location of tracheal lymphomas, hemoptysis is uncommon. Airway obstruction has been reported in 87% of these patients, with half of them requiring emergency intervention [11].

Chest X-ray is commonly obtained as part of the initial radiological investigation but is rarely diagnostic, and tumors may be easily missed. CT is the most useful imaging modality for the assessment of tracheal tumors and is the gold standard imaging technique for the diagnosis and evaluation of tumor extent. Bronchoscopy is usually performed to diagnose and stage tracheal tumors as well as obtaining tissue samples.

Reports in the literature of relapsing lymphoma involving the trachea are exceedingly rare. To date there have been 4 published cases [6–9] summarized in Table 1 (the present case is the fifth). These included 4 females and 1 male. The histology of three cases was Hodgkin's lymphoma and of two cases was non-Hodgkin's lymphoma. Duration between treatment and relapse ranged from 6 months to 4 years. Tracheal obstruction was found endoscopically in 4 of the 5 cases. Management of relapse varies from surgery alone, such as electrocautery or resection of tumors, to the addition of chemotherapy and radiotherapy. To our knowledge, the present case is the second case reported in the literature with relapsing non-Hodgkin's lymphoma involving the trachea. This is the first case reported in the current literature presenting with an acutely threatened airway requiring immediate surgical tracheostomy.

It is highly unusual for relapsing lymphoma to involve the trachea causing tracheal obstruction. Despite its rarity, it can present with life-threatening airway obstruction which may be rapidly progressive requiring immediate surgical intervention such as tracheostomy. Clinicians should be aware of this possibility, as our case necessitated prompt securing of the patient's airway.

## Figures and Tables

**Table 1:** Case reports of relapsing lymphoma involving trachea.

Case report	Sex	Histology	Symptoms	Duration between treatment and relapse (years)	Tracheal obstruction	Treatment of relapse	Outcome
Current study	F	NHL	Stridor, dyspnea, cough, hemoptysis	1/2	Y	S, C, R	Rapid regrowth of tumor, tracheotomized
Boujaoude et al., 2012 [6]	F	HL	—	4	Y	S	Relief of symptoms
Erturan et al., 2004 [7]	F	HL	—	3	Y	S	Relief of symptoms
Martin et al., 2011 [8]	M	HL	Dry cough, fever	1	N	S, C	Relief of symptoms
Ho et al., 1985 [9]	F	NHL	Dyspnea, wheezing	1	Y	S, C	No recurrence

M: male; F: female; HL: Hodgkin's lymphoma; NHL: non-Hodgkin's lymphoma; Y: yes; N: no; S: surgery; C: chemotherapy; R: radiotherapy.
